# Family planning practice and predictors of risk of inconsistent condom use among HIV-positive women on anti-retroviral therapy in Cambodia

**DOI:** 10.1186/1471-2458-14-170

**Published:** 2014-02-17

**Authors:** Naomi Nakaie, Sovanna Tuon, Ikuma Nozaki, Fuzuki Yamaguchi, Yuri Sasaki, Kazuhiro Kakimoto

**Affiliations:** 1 Graduate School of Nursing, Osaka Prefecture University 3-7-30, 583-8555 Habikino-city, Osaka, Japan; 2 National Maternal and Child Health Center, 31A, Rue de France Street, Phnom Penh, Cambodia; 3 Bureau of International Medical Cooperation, National Center for Global Health and Medicine, 1-21-1 Toyama, 162-8655 Shinjuku-ku, Tokyo, Japan; 4 Graduate School of Nursing, Osaka City University, 1-5-17 Asahimachi, 545-8585 Abeno-ku, Osaka, Japan; 5 Graduate School of Nursing, Nagoya City University, Kawasumi 1, Mizuho-ku, 467-8601 Nagoya-shi, Aichi, Japan

**Keywords:** HIV, ART, PMTCT, Family planning, Cambodia

## Abstract

**Background:**

In Cambodia, while anti-retroviral therapy (ART) services are increasingly available, the unmet needs of family planning among general population are high. These facts raise concern on possible exposure of many HIV-positive women on ART to the potential risk of unintended pregnancy. This study aimed to clarify family planning practices in Cambodia and determine predictors of risk of inconsistent condom use among women on ART.

**Methods:**

A cross-sectional survey with a structured questionnaire was conducted at five government-run health centers in Phnom Penh, Cambodia, from June to September, 2012. Multiple logistic regression analysis was used to identify predictors of risk of inconsistent condom use among regular users of contraceptive methods.

**Results:**

Of 408 respondents, 40, 17 and 10 used the pill, IUD, and injection, respectively, while 193 used condoms. 374 were not planning to have a child. Among 238 sexually active women who were not planning to have a baby, 59 were exposed to the risk of unintended pregnancy. Multivariate logistic regression analysis that did not include variables related to partners identified "seeking family planning information" (adjusted odds ratio (AOR): 2.6, 95% confidence intervals (95% CI): 1.1-6.2), awareness of mother-to-child transmission (MTCT) (AOR: 4.7, 95% CI: 1.9-11.6) and "having a son" (AOR: 2.0, 95% CI: 1.1-3.9) were significant predictors of inconsistent condom use. Another model that included all variables identified “able to ask a partner to use condom at every sexual intercourse” was the only predictor (AOR: 23.7, 95% CI: 5.8-97.6).

**Conclusions:**

About one-quarter of women on ART are at risk to unintended pregnancy although most do not plan to get pregnant. Furthermore, women on ART could be more empowered through improvement of communication and negotiation skills with partners to demand the use of condom during sexual intercourse. The use of other contraceptive methods that do not need partner involvement should be promoted.

## Background

The estimated number of people carrying HIV (PLHIV) was 34 million worldwide at the end of 2010 [1]. This number is increasing due to the recent reduction in mortality rate based on the availability of new and effective anti-retroviral therapy (ART) around the world, including poor and developing countries, where coverage of adults in need of ART was approximately 51% at the end of 2010 [2]. While the overall health status and life expectancy of PLHIV have improved with the use of ART, it has been reported that more HIV-positive women in African countries became pregnant after receiving ART [3-7].

In Cambodia, the scaling up of ART services has been so rapid that coverage of adults in need of ART is reported to have reached 94% in 2010 [8]. This coverage, which is considerably higher than in other Southeast Asian countries, is largely due to the efforts of the Health Ministry of the Cambodian Government. However, unmet needs for family planning among the general population of Cambodia in 2010 was as high as 16.6% [9,10]. This leads to the concern that many HIV-positive women on ART in Cambodia could be exposed to the potential risk of unintended pregnancy. In 2010, the national PMTCT (Prevention of Mother-to-Child Transmission) program in Cambodia reported a high ratio of HIV-positive mothers on ART among HIV-positive mothers at delivery, which suggested that many HIV-positive mothers become pregnant after starting ART [11,12]. Furthermore, it can be speculated that a proportion of these pregnancies would be unintended because of the reportedly high level of unmet family planning needs among the general population. Unintended pregnancy arises from the result of nonuse or incorrect use of contraceptives, or a noticeable contraceptive failure. It is reported that one of the major causes of unintended pregnancy is inconsistent contraceptive use [13]. Moreover, such practice can increase maternal mortality due to unsafe abortion and the risk of sexually-transmitted infections. Prevention of unintended pregnancies among HIV-positive women is included as a prong of a strategy for the PMTCT of HIV and considered increasingly important [14]. Thus, it is important to explore family planning practice among HIV-positive women on ART in Cambodia. The purpose of this study was to clarify family planning practices and determine predictors of risk of inconsistent condom use among Cambodian women on ART.

## Methods

A cross-sectional survey with a structured questionnaire was conducted at five government-run health centers, which were selected to cover a wide geographical area in Phnom Penh, the capital of Cambodia, from June to September in 2012. All 408 of the HIV-positive women aged 18-49 years on ART who visited these health centers during the study period were given information on the study, including its objectives and were offered the chance to participate. After obtaining written informed consent, face-to-face interviews with a structured questionnaire were administered to all 408 participants by trained interviewers at each health center in the Khmer language (the most widely spoken language in Cambodia). The questionnaire covered socio-demographic data such as age, marital status, education, number of children, duration on ART, awareness of mother-to-child-transmission (MTCT) people living together and disclosure of own HIV status and partner’s HIV status. Participants were asked about their pregnancy experience after the diagnosis of HIV, pregnancy planning, timing of their most recent sexual activity, partner and family planning methods in use during the most recent sexual vaginal intercourse, knowledge of family planning and negotiation with their partner in the employment of family planning methods. Awareness about MTCT was measured by asking three questions about modes of mother-to-child transmission, including: “1) during pregnancy”; “2) during delivery”; and “3) through breast feeding”. Those women who correctly answered all questions were regarded as being aware of MTCT.

**Figure 1:**
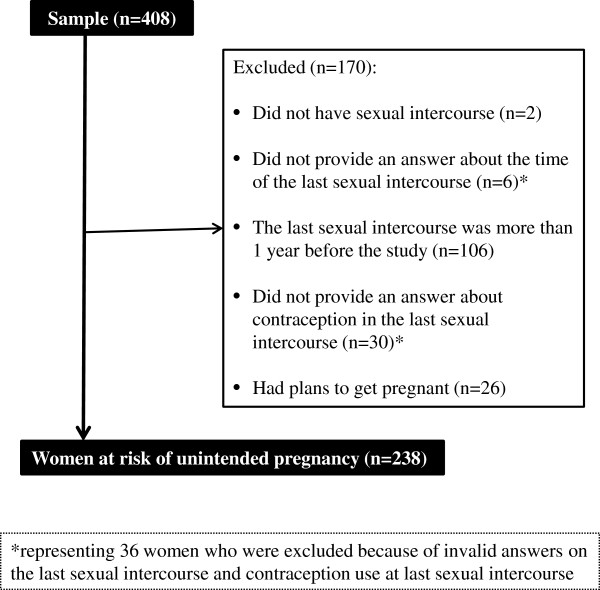
Details of the subject recruitment process.

In order to analyze factors associated with unintended pregnancy, only respondents who did not plan to have a baby were included in the analysis. Respondents who did not completely answer the question on contraceptive methods used in the most recent sexual intercourse, those whose last sexual intercourse was more than one year before the questionnaire and those who had never have sexual intercourse were excluded from the analysis. In total, 170 women were excluded from the analysis, leaving a sample population of 238 (Figure 1). Women at risk for unintended pregnancy were defined as sexually active women who were using modern contraception inconsistently or using an ineffective method of contraception. Inconsistent contraceptive use was defined with the question “Was a contraceptive method used every time you had sexual intercourse?”. Ineffective contraception was determined when the woman did not use any modern contraceptive method, such as condom, pill, intrauterine device (IUD) and injection in the most recent sexual intercourse. Therefore, of the above 238 women, those who did not use any modern contraceptive method at every sexual intercourse were perceived as being at risk of unintended pregnancy. The chi-square test was used to analyze differences in all variables between the two groups and the association of possible risk factors were estimated by odds ratio (OR) and 95% confidence intervals (95% CI). All independent variables with significant difference (*p* < 0.05) were selected and included in a multivariate logistic regression analysis. Data were processed and analyzed by IBM SPSS Statistics 20.0 for Windows.

Ethical clearance and approval for the study were obtained from the National Ethics Committee for Health Research, the Ministry of Health, Cambodia, as well as from the Research Ethical Committee of School of Nursing, Osaka Prefecture University.

## Results

### Socio-demographic data

Four hundred and eight HIV-positive women on ART who visited our study centers during the study period were invited to participate in the study, and all agreed to participate. The baseline characteristics, including the socio-demographic and partner-related variables are summarized in Table 1. The median age of participants was 37 years, and the median duration of ART was 32 months. Among the respondents, 243 (59.6%) were classified as married or living together with a partner; 48 (11.8%) were divorced or separated; 112 (27.5%) were widowed; and 5 had never married. The mean number of children was 2.2, and 277 of the women (67.9%) had a son. The number of women who had experienced pregnancy after HIV diagnosis was 159 (39.0%). Among all respondents, 237 (58.1%) lived with their partner. Of these partners, 192 (74.1%) were seropositive.

**Table 1:** Socio-demographic data (n = 408)

**Variables**	**n**	**%**
Socio-economic variables		
Age (median: 37 years)		
<37	197	48.3
≥37	211	51.7
Marital status		
Married or living together	243	59.6
Divorced or separated	48	11.8
Widowed	112	27.5
Never married	5	1.2
Health status		
Good	263	64.5
Some concerns	115	28.3
Feel bad	28	6.9
Education		
None	146	36.1
Primary	173	42.8
Secondary or higher	85	21.0
Number of children (mean: 2.2)		
Having a son	277	67.9
Having a daughter	247	60.5
Pregnancy experience after HIV diagnosis		
Yes	159	39.0
No	249	61.0
Awareness of MTCT		
Correct	369	90.4
Incorrect	39	9.6
Time since diagnosis of HIV positivity (median: 48 months)		
<48	202	49.5
≥48	186	45.6
Time on anti-retroviral therapy (median: 32 months)		
<32	195	47.8
≥32	198	48.5
Partner-related variables		
Living with partner		
Yes	237	58.1
No	171	41.9
Disclosed HIV status to partner		
Yes	237	58.2
No	170	41.8
Partner's HIV status^*^		
Positive	192	74.1
Negative	58	22.4
Unsure	9	3.5

^*^Total number of women with a partner. MTCT: mother to child transmission of HIV.

### Family planning practice of women on ART

Of the 408 women on ART, 374 (92.3%) were not planning to get pregnant (Table 2). Although the majority (398, 97.8%) of women were aware of at least one modern contraceptive method, only 194 (68.5%) responded that they used any contraceptive method at every sexual intercourse. With regard to the source of information on family planning, 356 (87.7%) received the information from a health facility such as a hospital, a health center, or a voluntary counseling and testing (VCT) center. In spite of this rate of access to family planning information, only 170 (58.6%) women reported were able to always ask their partners to use a condom and 175 (68.1%) were able to refuse sexual activity with partners. Awareness about the pill, IUD, injection and condoms as modern contraceptive methods was declared by 341 (83.8%), 249 (61.2%), 299 (73.5%) and 363 (89.2%) participants, respectively. However, those who actually used the pill, IUD and injection were 40 (14.2%), 17 (6.0%) and 10 (3.6%), respectively; although partners of 193 (68.7%) women used condoms. forty nine (17.5%) employed a dual contraceptive method including a condom plus another modern contraceptive. One hundred and fifty four (37.8%) had sexual intercourse once every four weeks, 37 (9.1%) every three months, 22 (5.4%) once a year, 113 (27.8%) once every more than one year, and two (0.5%) never had sexual intercourse.

**Table 2:** Family planning practice (n = 408)

**Variables**	**n**	**%**
Planning pregnancy		
Yes	27	6.7
No	374	92.3
Unsure	4	1.0
Knowledge of family planning methods (multiple answers)		
Any modern method	398	97.8
Sterilization	185	45.5
Pill	341	83.8
Intrauterine device	249	61.2
Injection	299	73.5
Implant	191	46.9
Condom	363	89.2
Any traditional method	0	0.0
None	9	2.2
Source of family planning		
Any health facility	356	87.7
Used contraceptive method during sexual intercourse^*^		
Every time	194	68.5
Sometimes/Never	62	29.5
Unsure	2	2.0
Being able to ask partner to use condom^*^		
Always	170	58.6
Sometimes	85	29.3
Never	35	12.1
Being able to refuse sexual intercourse with partner^*^		
Possible	175	68.1
Impossible	70	27.2
Unsure	12	4.7
Variables related to last sexual intercourse		
Time		
≤4 weeks	154	37.8
≤3 months	37	9.1
≤1 year	22	5.4
>1 year	113	27.8
Never	2	0.5
Unsure	79	19.4
Partner		
Husband	223	87.7
Partner living together	15	5.9
Boyfriend	10	3.9
Other	1	0.4
Unsure	5	2.0
Family planning methods (multiple answers)		
Any modern method	228	81.4
Sterilization	9	3.2
Pill	40	14.2
IUD	17	6.0
Injection	10	3.6
Implant	9	3.2
Condom	193	68.7
Any traditional method	3	1.1
Dual method (condom + modern method)	49	17.5
None	10	15.2

^*^Total number of women with partners.

### Variables associated with the risk of inconsistent condom use

Next, the predictive factors of the risk of inconsistent condom use were analyzed using data of 238 women who met the inclusion criteria (Figure 1). The number of women exposed to the risk of unintended pregnancy, i.e., women who answered “not using modern contraceptives every sexual intercourse” was 59 (24.8%) (Table 3). Bivariate analysis identified the following variables that were significantly associated with a lower risk of inconsistent condom use: having a son (*p* = 0.029), pregnancy experience after HIV diagnosis (*p* = 0.043), awareness of MTCT (*p* < 0.001), living with a partner (*p* = 0.001), disclosed of HIV status to a partner (*p* < 0.001), HIV-positive partner (*p* = 0.032), obtaining family planning information from health facility (*p* = 0.012), being able to ask a partner to use condom at each sexual intercourse (*p* < 0.001), being able to refuse a sexual intercourse with a partner (*p* < 0.001), and the partner at the last sexual intercourse was a husband (*p* = 0.001). Age, education or having a daughter were not associated with a lower risk of inconsistent condom use.

**Table 3:** **Associations between routine use of modern contraceptive method and related factors: Multivariate logistic regression showing adjusted odds ratio’s (AOR) and 95**% **confidence intervals [95**% **CI]**

**Variables**	**Using modern contraceptives every time**			**p value**	**Adjusted odds ratio**	
	**Total (n=238) [%]**	**Yes (n=179) [75.2%]**	**No (n=59) [24.8%]**		**Model 1: women status**	**Model 2: all variables**
Socioeconomic variables						
Age (median: 37 years)						
<37	138[58.0]	103[74.6]	35[25.4]	0.810	―	―
≥37	100[42.0]	76[76.0]	24[24.0]			
Education						
Less than primary	189[80.1]	140[74.1]	49[25.9]	0.510	―	―
Secondary or Higher	47[19.9]	37[78.7]	10[21.3]			
Duration of ART (median: 32 months)						
<32	48.5	86[75.4]	28[24.6]	0.878	―	―
≥32	51.5	85[74.6]	29[25.4]			
Having a son						
No	70[29.4]	46[65.7]	24[34.3]	0.029	1.0	1.0
Yes	168[70.6]	133[78.7]	35[21.3]		2.0^**[^1.1-3.9]	2.2[0.9-5.9]
Having a daughter						
No	89[37.4]	63[70.8]	26[29.2]	0.222	―	―
Yes	149[62.6]	116[77.9]	33[22.1]			
Pregnancy experience after HIV diagnosis						
No	124[52.1]	100[80.6]	24[19.4]	0.043	1.0	1.0
Yes	114[47.9]	79[69.3]	35[30.7]		0.6[0.3-1.1]	1.2[0.4-3.8]
Awareness of MTCT						
Correct	215[90.3]	169[78.6]	46[21.4]	<0.001	4.7^**^[1.9-11.6]	2.1[0.5-9.9]
Incorrect	23[9.7]	10[43.5]	13[56.5]		1.0	1.0
Partner-related variables						
Living with a partner in same house						
Yes	211[88.7]	166[78.7]	45[21.3]	0.001	―	8.1[0.6-113.9]
No	27[11.3]	13[48.1]	14[51.9]			1.0
Disclosed HIV status to a partner						
Yes	207[87.0]	166[80.2]	41[19.8]	<0.001	―	2.0[0.3-13.9]
No	31[13.0]	13[41.9]	18[58.1]			1.0
Partner's HIV status						
Negative	52[21.8]	36[69.2]	16[30.8]	0.032	―	1.0
Positive	159[66.8]	132[83.0]	27[17.0]			0.8[0.3-2.4]
Family planning-related variables						
Obtaining family planning information from health facility						
No	27[11.3]	15[55.6]	12[44.4]	0.012	1.0	1.0
Yes	211[88.7]	164[77.7]	47[22.3]		2.6^*^[1.1-6.2]	2.1[0.6-7.7]
Being able to ask a partner to use condom at each sexual intercourse						
Possible	151[63.4]	146[96.7]	5[3.3]	<0.001	―	23.7^***^[5.8-97.6]
Impossible	84[35.3]	33[39.3]	51[60.7]			1.0
Being able to refuse a sexual intercourse with a partner						
Possible	160[69.9]	143[89.4]	17[10.6]	<0.001	―	2.1[0.7-6.5]
Impossible	69[30.1]	33[47.8]	36[52.2]			1.0
Person in last sexual intercourse was a husband						
Yes	203[86.8]	162[79.8]	41[20.2]	0.001	―	3.2[0.7-15.1]
No	31[13.2]	16[51.6]	15[48.4]			1.0

***p<0.001, **p<0.01, *p<0.05 MTCT: mother to child transmission of HIV.

### Multivariate logistic regression analysis to determine associations of risk of inconsistent condom use

Two multivariate logistic regression models were employed in the analysis of data (Table 3). In model 1, which excluded partner-related variables, obtaining family planning information from health facilities (adjusted odds ratio (AOR): 2.6, 95% CI: 1.1-6.2, *p* < 0.05), awareness of MTCT (AOR: 4.7, 95% CI: 1.9-11.6, *p* < 0.01), and having a son (AOR: 2.0, 95% CI: 1.1-3.9, *p* < 0.05) were identified as significant predictors of the use of modern contraceptive methods at every sexual intercourse. Model 2, which included all variables, identified being able to ask a partner to use of condom as the only significant predictor of the use of modern contraceptive methods at every sexual intercourse (AOR: 23.7, 95% CI: 5.8-97.6, p < 0.001). Data of 38 respondents were excluded from the bivariate analysis and logistic regression analysis due to invalid answer about the last sexual intercourse (Figure 1). Women who were widowed were 75.0% despite 27.5% among all respondents. The percentages of education and health status indicated a similar tendency of all respondents (Table 4).

**Table 4:** Socio-demographic data (n = 36^*^)

**Variables**	**n**	**%**
Age (median: 37 years)		
<37	22	61.1
≥37	14	38.9
Marital status		
Married or Living together	4	11.1
Divorced or Separated	5	13.9
Widowed	27	75.0
Never Married	0	0
Education		
None	7	19.4
Primary	19	52.8
Secondary or Higher	10	27.8
Health status		
Good	18	50.0
Some concerns	15	41.7
Feel bad	3	8.3
Time since diagnosis of HIV positivity (median: 48 months)		
<48	23	63.9
≥48	12	33.3
Unsure	1	2.8

^*^36 women who were excluded due to invalid answers regarding the last sexual intercourse and contraception use at last sexual intercourse.

## Discussion

Although previous studies reported that HIV-positive women tend to plan to have a baby because of their personalized stigma [15-17], our study that targeted HIV-positive Cambodian women on ART showed that the majority (92.3%) of respondents were not planning to have a child, although some reported the experience of losing a child due to MTCT of HIV and surrounding pressure to have a baby. The difference between this study and the above previous studies may be due to differences in fertility preference, given that the median age of respondents was 37 years, or because more than 80% of women of this study had at least one child. Our results demonstrated that many (61.2-89.2%) of our respondents had knowledge of condoms, the pill, injection and IUD as modern contraceptive methods and that the majority (68.7%) were actually using condoms. The percentage of women using other modern methods ranged from 3.2 to 14.2%. This means that the proportions of women using the pill, IUD or injection were relatively low compared with the level of awareness about these methods. According to the Cambodia Demographic and Health Survey, 2010 [9], 95% of women of the general population of Cambodia have some knowledge about condoms, the pill, injection, or IUD as family planning methods, and the pill is the most commonly used method of family planning (9.5%), with only 1.7% reporting the use of condoms [9]. This was considerably different to the women from our study, all of whom were on ART. Our results indicated that women on ART were much more inclined to use condoms for family planning compared to women in the general population. Interestingly, the proportion of women on ART using condoms in Cambodia seems much higher than that in African countries, where the rates ranged from 30 to 50% [18]. This means that Cambodian HIV female carriers seem to be better educated on condom use as a means of preventing HIV transmission to their partners as well as for avoiding unintended pregnancy. However, since the choice of contraceptive method for family planning among HIV-positive women seems to be limited to condoms, which requires cooperation from the male partner, it might be difficult for such women to achieve their desired fertility choice.

Our study also demonstrated that approximately one quarter of the respondents who were not planning to have a child was exposed to the risk to unintended pregnancy by not using any modern contraceptive method. Unintended pregnancy can lead to unsafe abortion, which is a major cause of maternal death in developing countries, such as Cambodia [4,6]. In addition, prevention of unintended pregnancy among HIV-positive women is stressed as the second prong for PMTCT. However, according to CDHS2010, the unmet need for family planning among the general population in Cambodia was 16.6%, which has recently been declining but is still high in comparison to other Southeast Asian countries, largely due to insufficient opportunities for women to obtain family planning information [9]. There is a need to emphasize that Cambodian women on ART should be better protected against unintended pregnancy, and that it is necessary to consider an effective strategy to reduce the risk by assessing predictors of the risk of unintended pregnancy. On the other hand, taking the male-dominant culture into account, multivariate logistic regression analysis was performed in the present study using two models. The first model focused on women-related predictors, with the expectation that individual support for each woman would be considered in further detail. The second model included all variables and assessed the predictors to the risk of inconsistent condom use.

Since multivariate regression model 1 identified seeking information on family planning from government health facilities was an independent predictor of routine use of modern contraceptive methods, effective methods should be considered to dissipate information on family planning. Our respondents, as HIV-positive women on ART, must be encouraged to visit health facilities periodically to obtain their medications, and this relation should be utilized to link ART centers and family planning services. To enhance motivation of HIV-positive women to family planning practice, it is likely that family planning services provided by medical personnel, including counselors, could eventually lead to a reduction in the risk of unintended pregnancy [4,19]. Alternatively, the health facilities at ART sites should also provide family planning services as part of the ART service, so that women do not have to disclose their HIV status to family planning services. Moreover, the timing of the provision information on family planning to women after starting ART needs to be taken into account. A study from South Africa reported that unmet family planning needs rose in the year following the beginning of ART [6]. In this regard, the provision of information soon after starting ART would seem unsuitable since HIV-positive patients likely receive a great deal of information at that time, including their health status and explanation regarding ART medications [4].

Another predictor of the use of modern contraceptive methods in Model 1 was awareness of MTCT. While there are no studies that have directly determined the association between awareness of MTCT and family planning practices, a study from Uganda reported that HIV-positive women who were aware of the short life expectancy of HIV-infected children tended to use contraceptive methods [20]. By being aware of MTCT, HIV-positive women might be more concerned about the chance of mothering HIV-infected children, and thus plan to have no more children [15]. Although providing information on MTCT seems to be an effective way to avoid unintended pregnancies, health care providers involved in family planning services, e.g., counselors, should be aware of the sensitivity of MTCT issue for HIV-positive women. Information on MTCT could have negative effects on family planning and fertility desires by promoting the recalling of a trauma, feeling of guilt in relation to the loss of a child due to MTCT, or anxiety regarding possible infection of a child or possibility that the child may become an orphan in the future [7,15,16,20]. Therefore health care providers should be careful while providing education on MTCT to women on ART; they should advise women on the need to reduce the risk of unintended pregnancy but at the same time avoid negative psychological effects.

Having a son was also a predictor for use of modern contraceptive methods. In our study, HIV-positive women who did not have sons were less likely to employ adequate contraceptive methods regardless of their family planning and fertility desire. Similar findings have also been described in several other studies [21-23]. Reports from India and Uganda have shown a preference among HIV-positive people in those countries for having sons [4,7]. In Cambodia, boys also tend to be preferred culturally and a disparity between boys and girls in education, nutrition, and health aspects has been reported [24]. Thus, the cultural background about the preference for male children should be further investigated.

In Model 2, which included all variables related to both HIV-infected women and their partners, only “Being able to ask a partner to use a condom” was a significant predictor related to the routine use of a modern contraceptive method. The lack of significance of the predictors identified in Model 1 by the logistic regression analysis could be related to possibility that the ability of negotiation for condom use confounded each significant variable. Since condom use was the main contraceptive method among our respondents, the ability to ask the partner to use condom is likely to be a key in preventing unintended pregnancy. Although the condom is an easy and effective tool with little side effects for contraception, this method requires the cooperation of the male partner, who may not always view its use positively based on interruption of sexual pleasure and sexual function or because the partner is the final decision maker and the woman request is refused [18]. Moreover, in the general population, there are some cultural norms or behaviors such as subordination, discrimination, and violence against women, with men tending to have more power to control sexual relationships [25-28]. This is reflected by the low proportion of women using condoms for family planning in Cambodia compared with those using methods that do not require the partner’s cooperation such as the pill, injection and IUD. Thus, although the ability to negotiate condom use was identified as a significant determinant in the prevention of inconsistent condom use in our study, similar to the findings of previous studies [12,29], cultural background should be carefully considered so that women in Cambodia who want to enforce the use of condoms for family planning may be empowered through improvements to communication and negotiation skills. A dual contraceptive method, i.e., condom plus a modern contraceptive which does not require the cooperation of the male partner, should be better promoted among HIV-infected women in order to protect them from both pregnancy and disease.

In our analysis, 36 women were excluded due to invalid answers about the last sexual intercourse and contraceptive use then, which might be sensitive questions to answer, and this exclusion could lead to a bias. However, many of the 36 excluded women were old, widowed or divorced, who might not have any stable partner, or they did not feel obligated to answer questions on family planning practice and details of sexual intercourse. Therefore, the exclusion of these 36 women from our analysis was unlikely to have biased our analysis.

### Study limitations

The present study had certain limitations. First, since this study was conducted in the capital of Cambodia, where access to health facilities was better than in rural areas, and the sampling of sites was purposive, it cannot generalize the study findings to the overall population. Moreover, since it was a health facility-based study, the respondents did not include women who have a little motivation to control their own health and rarely visit health facilities. These women might be at greater risk with other predictors of unintended pregnancy. Second, the structured questionnaire based on a self report may be subject to recall bias; thus, there is possibility of being imprecise or false answers with the actual family planning practice. However, respondents whose last sexual intercourse was more than one year before the questionnaire were excluded from the analysis to avoid potential bias. Third, the odds ratio was noticeably large because the ability of negotiation for condom use was strongly related to some predictors, potentially creating imbalance in cell size due to the relatively small population sample. Despite these limitations, our selection of five study sites among 11 in Phnom Penh and our quantitative analysis mean that our findings likely represent the reality of family planning practice among HIV-positive women on ART in Cambodia. To our knowledge, this is the first study implying important issues and suggestions in order to prevent unintended pregnancy among women on ART, in Southeast Asia.

## Conclusions

The present study showed that nearly one quarter of the investigated women were at risk of unintended pregnancy, suggesting a high unmet need for pregnancy among HIV-positive women on ART in Cambodia. These women on ART mainly use condom in their family planning compared with methods that do not require male cooperation. The results suggest that the ability of women on ART to enforce condom use was a significant predictor and that they could be better empowered through improvements of communication and negotiation skills to help them achieve this end. Furthermore, the results suggested that, in order to prevent unintended pregnancy, male partners should be more involved in family planning. Otherwise, in order to help prevent unintended pregnancies, health professionals providing family planning information to women on ART need to promote a dual method, including a method that does not require male cooperation.

## Abbreviations

ART: Anti-retroviral therapy; IUD: Intrauterine device; MTCT: Mother to child transmission of HIV; PLHIV: People living with HIV; PMTCT: Prevention of mother to child transmission of HIV; VCT: Voluntary counseling and testing.

## Competing interests

The authors declare that they have no competing interest.

## Authors’ contributions

NN, IN, FY, YS and KK carried out data analysis and drafted the manuscript. NN, TS, FY and KK helped collect the data and participated in coordinating the study design to involve trained interviewers. TS, IN, FY, YS and KK helped with the design of the study. All authors read and approved the final manuscript.

## Pre-publication history

The pre-publication history for this paper can be accessed here:

http://www.biomedcentral.com/1471-2458/14/170/prepub
